# Paradoxical Immune Reconstitution Syndrome Presenting as Acute Respiratory Distress Syndrome in a Leukemia Patient during Neutrophil Recovery

**DOI:** 10.1155/2012/670347

**Published:** 2012-06-11

**Authors:** Issam A. Alawin, Bernard M. Karnath

**Affiliations:** Internal Medicine Department, The University of Texas Medical Branch at Galveston, Galveston, TX 77555, USA

## Abstract

Immune reconstitution inflammatory syndrome (IRIS) in the setting of antiretroviral therapy is well described, but it is not as common in non-HIV patients; here, we present a case of immune reconstitution inflammatory syndrome presenting as acute respiratory distress syndrome in a leukemia patient who had neutropenic fever and septic shock after high-dose cytarabine. During neutropenia recovery, his chest X-ray showed progressive worsening despite being on adequate therapy, we started him on steroids which resulted in significant clinical improvement.

## 1. Clinical Vignette

**Figure 1:**
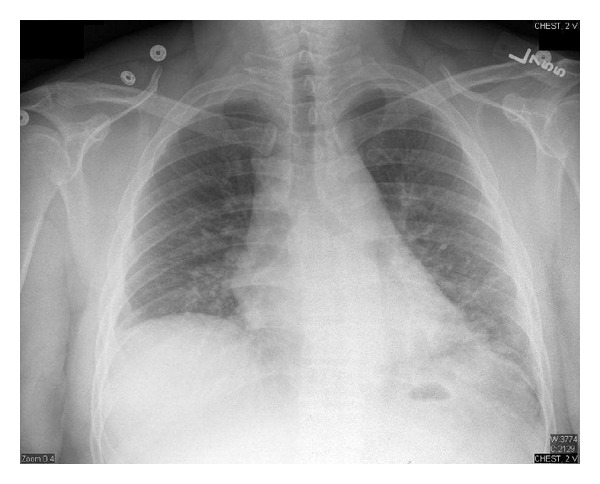
Chest X-ray on day 14 after chemotherapy (ANC was less than 100 cell/*μ*L).

A 42-year-old male with acute myeloblastic leukemia (FAB classification AML M2, WHO classification: AML with recurrent genetic abnormalities: t (8 : 21)) received induction chemotherapy with cytarabine and idarubicin twice (he did not achieve remission with the first induction) followed by consolidation with high-dose cytarabine. He received three cycles of high-dose cytarabine without incident. He was admitted to receive his fourth cycle and did well until fourteen days after chemotherapy when he developed neutropenic fever. His maximum temperature was 40.1°C, his total white blood cell count was less than 100 cells/*μ*L, blood cultures were drawn, and a chest X-ray was done (Figure 1). He was started on piperacillin/tazobactam and vancomycin. The next day, the two blood culture sets grew Gram-positive cocci in chains which were identified later to be streptococcus viridans. The same day, he became hypotensive and hypoxic (he required 100% FiO_2_ by nonrebreathing mask to maintain SpO_2_ above 95%), he was transferred to the intensive care unit for septic shock and acute hypoxic respiratory failure, and he required vasopressors and intubation for mechanical ventilation. Patient was started on caspofungin.

Over the following three days, he continued to have low-grade fever but overall the temperature was trending down and his oxygen requirement represented by FiO_2_ and positive end expiratory pressure (PEEP) were weaned down. We did a bronchoscopy looking for a cause of infection and hypoxia, bronchoalveolar lavage (BAL) grew respiratory flora, and there was no evidence of aspergillus.

**Figure 2:**
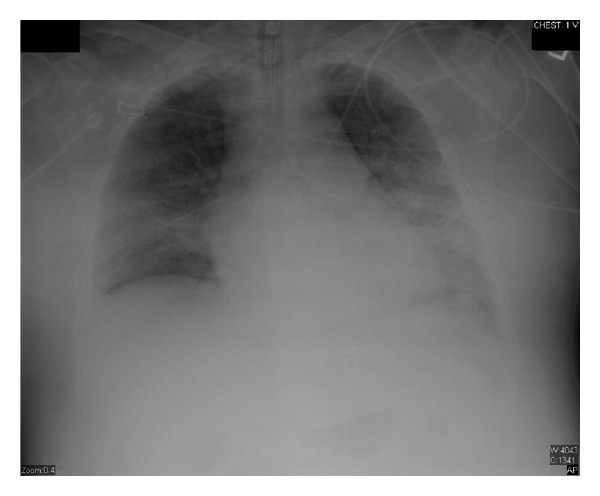
Chest X-ray day 18 after chemotherapy (ANC was 1100 cell/*μ*L).

**Figure 3:**
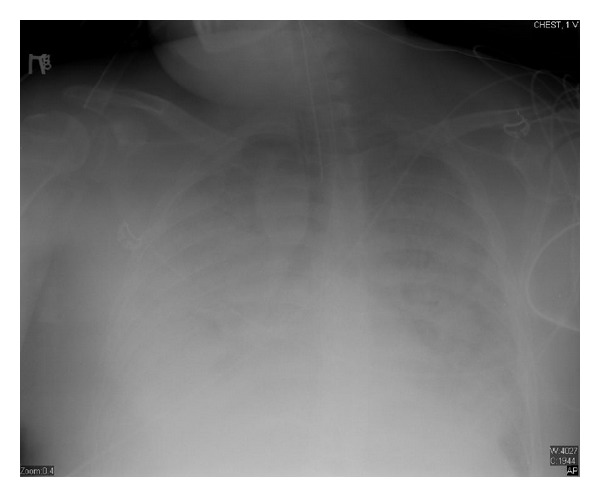
Chest X-ray day 22 after chemotherapy (ANC was 11,500 cell/*μ*L).

Eighteen days after chemotherapy, the day of recovery of neutropenia, his CXR showed significant worsening with bilateral infiltrates (Figure 2), his fever trended up, and his mechanical ventilation settings (FiO_2_ and PEEP) increased, he developed acute respiratory distress syndrome (his PaO_2_/FiO_2_ was less than 200 with bilateral infiltrates on CXR). Over the following three days, he continued to deteriorate clinically and CXR was getting worse (Figure 3) despite being on appropriate antibiotics and without an evidence of a new infection. Granulocyte colony-stimulating factor (GCSF) was discontinued when the absolute neutrophilic count reached 8000 cell/*μ*L. A transthoracic echocardiogram did not show an evidence of heart failure or valvular vegetation.

Three days after his neutrophil recovery, we concluded that his deterioration must be attributed to the recovering immune system, the diagnosis of paradoxical immune reconstitution syndrome was made, and we started him on methylprednisolone 125 mg IV q 6 hours. Within 48 hours, his fever trended down and we were able to start weaning his oxygen requirements despite no significant change in his CXR (actually CXR did not show a significant change until 10 days later).

**Figure 4:**
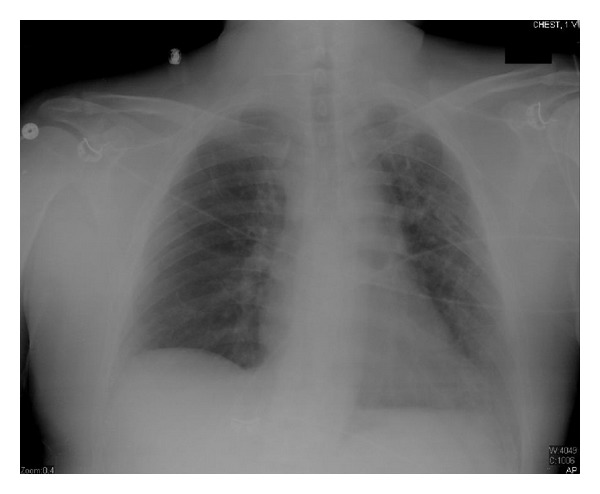
Chest X-ray day 31 after chemotherapy (ANC was 6700 cell/*μ*L).

After one week, we tapered his steroids down to prednisone 40 mg daily, antibiotics were continued for two weeks, patient was extubated successfully after 18 days of intubation, and CXR day 31 after chemotherapy is shown (Figure 4). He was transferred to the floor, and physical therapy was started. Prednisone was gradually tapered to continue a total period of three weeks of steroids therapy. Patient was discharged to a medical facility to continue occupational and physical therapy.

## 2. Discussion

### 2.1. Definition

Immune reconstitution inflammatory syndrome is an exaggerated and dysregulated inflammatory response to invading microorganisms. It manifests when a rapid shift of host immunity from an anti-inflammatory and immunosuppressive state towards a pathogenic proinflammatory state occurs. Immune reconstitution inflammatory syndrome gained increased recognition in HIV patients receiving antiretroviral therapy (ART).

IRIS manifests in one of two forms: unmasking IRIS occurs as a result of reconstitution of adaptive or innate immunity to an antigen or pathogen, with the disease or infection becoming clinically apparent. In contrast, paradoxical IRIS denotes a worsening of an already diagnosed clinical disease which applies to our patient.

Incidence, risk factors, clinical features, and management of neutrophil recovery-associated IRIS are not as well published as in HIV-associated IRIS; therefore, throughout the discussion of our case, we will compare and contrast clinical data between these two entities.

### 2.2. Epidemiology

IRIS was described before the HIV epidemic in patients receiving therapy for tuberculosis [1], but it became much more evident after the introduction of antiretroviral l therapy (ART) during the last decade of the twentieth century, according to a large multisite US HIV-infected outpatient cohort, IRIS occurred in 10.6% of patients who responded to effective ART and contributed to increased mortality [2]. In another study, Shelburne et al. found the incidence of IRIS of any type to be 32% in patients during ART [3].

IRIS is also observed in non-HIV immunocompromised patients as solid organ transplantation recipients, postpartum period, tumor necrosis factor (TNF) antagonist recipients, and as in our case hosts during neutrophil recovery after chemotherapy [4].

Risk factors for IRIS in HIV patients include low CD4 and high viral load at the time of effective ART initiation or resumption [2], duration of immunodeficiency, velocity and extent of immune recovery, and genetic susceptibility (distinct HLA types and polymorphisms in cytokine genes) [5]. Patients with hematological malignancies after high-dose chemotherapy develop immune reconstitution due to recovery of the innate immunity [6], the most crucial risk factor for IRIS in this category is the velocity of recovery of neutrophil count [7], the latter one was clearly observed in our patient as his ANC increased 10-folds within four days (from 1100 cell/*μ*L on day 18 to 11,500 cell/*μ*L on day 22 post chemotherapy), and GCSF was only discontinued when it reached 8,000 cell/*μ*L. This demonstrates the importance to understand the balance between GCSF benefits to restore immunity versus the potential harm in developing IRIS during neutrophil recovery.

### 2.3. Clinical Manifestation

Presentation of IRIS usually correlates with the organ involved by the opportunistic infection; manifestations include fever, skin nodules, ocular lesions, bone and joint involvement, liver lesions, bladder lesions, and lung infiltrates.

Pulmonary IRIS in particular was thoroughly described, especially in the setting of TB infection, symptoms can range from cough, wheezes, worsening chest X-ray to respiratory failure [8, 9], and acute respiratory distress syndrome [10, 11].

IRIS presenting as ARDS was first reported by Goldsack et al. [10] in an HIV patient with pulmonary tuberculosis. Wikman et al. [11] described a non-HIV patient who suffered from ARDS as a manifestation of IRIS secondary to pulmonary TB; in our patient, ARDS was a systemic manifestation of IRIS secondary to streptococcus viridans septicemia rather than an exaggerated response against a pulmonary infection.

In patients recovering from neutropenia, IRIS tends to happen up to 15 days after the resolution neutropenia (mean of 2 days) [8]; in our patient, the clinical and radiological deteriorations were noticed on the first day of neutropenia resolution, whereas HIV-associated IRIS appears to develop 8–150 days (mean of 33 days) after starting ART [12].

### 2.4. Diagnosis

Diagnosis of IRIS is not straightforward as there are no universal clinical criteria or diagnostic tests to confirm it. In our case, we initially attributed the ARDS to sepsis but when we noticed the initial relative response, the strong temporal relationship between deterioration and the neutrophil recovery, the negative BAL culture, and repeatedly negative blood cultures, we decided to make the diagnosis.

Clinical deterioration in similar cases can also be due to progression of the current opportunistic infection, failure of therapy, a new infection, or drug interaction. Different studies provided a number of different clinical criteria to help diagnose IRIS. In general, these criteria include the following [8]:

1. temporal association between neutrophil recovery and clinical deterioration;
2. robust microbiologic response (no evidence of worsening infection);
3. subsequent resolution without antimicrobial modification;
4. improved outcome after immunomodulation using medications like steroids.

Whether ARDS in our patient was solely caused by IRIS or the combined effect of septic shock and IRIS might never be known.

### 2.5. Management

Treatment of IRIS in the setting of recovery from neutropenia is difficult as it largely depends on case reports, even in HIV-associated IRIS, there is only one randomized placebo-controlled trial of prednisone for the treatment of paradoxical TB-IRIS [13]. In this trial which included 110 participants, Meintjes et al. found significant improvement in symptoms and quality of life in patients who received prednisone compared to those who received placebo, and it is interesting that the timeline for chest X-ray improvement was 2–4 weeks exactly like our patient. Prednisone dose was 1.5 mg/kg/day for two weeks followed by half the dose for another two weeks. As was seen in our patient, it is also important to withdraw GCSF when IRIS is suspected.

## 3. Conclusion

Immune reconstitution inflammatory syndrome is associated with increased morbidity and mortality; it can be a medical mystery as it depends largely on high suspicion and ruling out other etiologies. A consensus about clinical criteria or a diagnostic laboratory test is needed to help diagnosing this disease, and more randomized controlled trials are needed to confirm the role of steroids.
